# 5-Methyltetrahydrofolate Attenuates Oxidative Stress and Improves Kidney Function in Acute Kidney Injury through Activation of Nrf2 and Antioxidant Defense

**DOI:** 10.3390/antiox11061046

**Published:** 2022-05-25

**Authors:** Charith U. B. Wijerathne, Kathy K. W. Au-Yeung, Yaw L. Siow, Karmin O

**Affiliations:** 1 St. Boniface Hospital Research Centre, Winnipeg, MB R2H 2A6, Canada; cwijerathne@sbrc.ca (C.U.B.W.); kauyeung@sbrc.ca (K.K.W.A.-Y.); 2 Department of Animal Science, University of Manitoba, Winnipeg, MB R2H 2A6, Canada; 3 Department of Physiology & Pathophysiology, University of Manitoba, Winnipeg, MB R2H 2A6, Canada; csiow@sbrc.ca; 4 Agriculture and Agri-Food Canada, St. Boniface Hospital Research Centre, Winnipeg, MB R2H 2A6, Canada

**Keywords:** acute kidney injury (AKI), oxidative stress, glutathione, ischemia-reperfusion, folic acid

## Abstract

Oxidative stress is a major mediator of adverse outcomes in acute kidney injury (AKI). Deficiency of micronutrients, such as folate, is common in AKI. Our previous study reported that AKI impaired kidney reabsorption of folate and decreased plasma folate level in rats. The present study investigated the effect of 5-methyltetrahydrofolate (5-MTHF), a biologically active form of folate/folic acid, on AKI-impaired kidney function and oxidative stress. Sprague-Dawley rats developed AKI after kidney ischemia (45 min) and reperfusion (24 h). Injection of 5-MTHF (3 µg/kg body weight) improved kidney function and attenuated oxidative stress with a restoration of glutathione and a reduction of lipid peroxidation in the kidney. Injection of 5-MTHF activated transcription factor Nrf2 and increased the expression of glutathione synthesizing enzymes, superoxide dismutase-1 and heme oxygenase-1 in the kidney. Simulated ischemia-reperfusion through hypoxia-reoxygenation increased oxidative stress in proximal tubular cells. Incubation of cells with 5-MTHF alleviated cell injury and increased antioxidant enzyme expression and intracellular glutathione levels. Inhibition of Nrf2 expression through siRNA transfection abolished the effect of 5-MTHF against oxidative stress. These results suggest that low-dose folic acid can improve kidney function through activation of Nrf2 and restoration of antioxidant defence. Micronutrient supplements may improve clinical outcomes in AKI.

## 1. Introduction

Folate is water-soluble vitamin B9 that plays an important role in one-carbon reactions that are essential for DNA synthesis and methylation reactions [1]. Folate is widely distributed in foods with dark green leafy vegetables and animal liver as the richest source of naturally occurring folate. Folic acid is the synthetic form of folate that is added to fortified foods and supplements. Following intestinal absorption, folate/folic acid is metabolized to 5-methyltetrahydrofolate (5-MTHF), a biologically active form of folate. Folate deficiency is linked to birth defects, cancer, hyperhomocysteinemia and metabolic disease [2,3,4,5]. Nutrient deficiency can perturb redox balance, which, in turn, exacerbates kidney injury [6].

Acute kidney injury (AKI) is characterized by a rapid loss of kidney function and is associated with high mortality rates [7,8]. Kidney ischemia-reperfusion is one of the most common causes of AKI [9]; it is a result of a sudden reduction or stop of blood flow to the kidney and subsequently a re-establishment of blood flow to the ischemic region. Ischemia-reperfusion elicits a multitude of cellular events ranging from the depletion of oxygen and nutrients, increased oxidative stress, inflammatory response, mitochondria injury to cell death, and ultimately causes organ damage [9]. Oxidative stress occurs when there is an imbalance between reactive free radical generation and antioxidant defences [10,11]. Restoration of blood flow exacerbates ischemia-induced hypoxic injury due to a rapid rise of oxygen-derived free radicals in the affected tissue, causing oxidative stress. Oxidative stress plays a key role in ischemia-reperfusion-induced kidney injury [12,13]. Micronutrient deficiency is common in patients with kidney diseases and is correlated to the high mortality of AKI.

Currently, there are no standard pharmacological agents clinically approved to treat AKI effectively. Early detection and prevention are the key strategies for AKI management [14,15]. According to a commentary on the *Kidney Disease: Improving Global Outcomes* (KDIGO) clinical practice guidelines for AKI, the supply of adequate nutrients plays a vital role in reducing metabolic complications in AKI patients [16]. Malnutrition is highly prevalent in AKI patients and is associated with an increased risk of morbidity and mortality [17]. Renal replacement therapy, such as dialysis and kidney transplantation, are the main treatment options for patients with end-stage renal disease; however, patients with renal replacement therapy continue to experience nutrient deficiency, oxidative stress, and damage to kidney tissue [18,19]. Many patients with kidney diseases have a deficiency of micronutrients including electrolytes, vitamins and trace elements [16,17,20]. Targeting oxidative stress has been implicated in improving clinical outcomes in patients with kidney transplantation [18]. Previous studies reported renal protective effects of antioxidants against ischemia-reperfusion-induced kidney injury [12,14,21]. Aside from its essential role in growth and development, folic acid also has an antioxidant effect including free radical scavenging and activation of cellular antioxidant defence [2,3,22,23,24]. Our previous studies showed that folic acid supplementation attenuated oxidative stress in rats and mice with diet-induced hyperhomocysteinemia and fatty liver [22,23,24].

The nuclear factor erythroid 2-related factor 2 (Nrf2) is a key regulator of the antioxidant defence system in mammalian cells. This transcription factor is activated in response to oxidative stress [25,26]. The activation of Nrf2 plays an essential role in restoring antioxidant defence against oxidative stress injury. Upon activation, Nrf2 is translocated to the nucleus where it binds the antioxidant response elements (ARE) in the promoter region of genes encoding enzymes that are involved in the antioxidant defence mechanism [25,26]. Activation of Nrf2 upregulates the expression of antioxidant enzymes including, heme oxygenase-1 (HO-1), superoxide dismutase (SOD) and glutathione synthesizing enzymes [26,27]. The HO-1 is a rapidly inducible cytoprotective protein in response to oxidative stress. Activation of Nrf2 robustly induces the HO-1 expression that mediates the immediate antioxidant response to various oxidative stress conditions including ischemia-reperfusion injury [28]. SOD is one of the major antioxidant enzymes that detoxify reactive oxygen species (ROS). Glutathione, a thiol-containing tripeptide, is a potent non-enzymatic antioxidant that regulates redox balance [29]. Glutamate-cysteine ligase catalyzes the rate-limiting reaction by converting cysteine and glutamate to *γ*-glutamyl-cysteine. This enzyme is composed of two protein subunits, namely, the glutamate-cysteine ligase catalytic subunit (Gclc) and modifier subunit (Gclm). The next reaction is catalyzed by glutathione synthetase that converts *γ*-glutamyl-cysteine and glycine to glutathione [29]. The reduced form of glutathione (GSH) is one of the major electron donors that neutralize reactive free radicals and subsequently oxidize to glutathione disulphide (GSSG) [29]. Patients with chronic kidney disease (CKD) had a low plasma GSH level and/or GSH to GSSG ratio [30,31]. We observed a significant reduction of GSH levels in the kidney tissue and plasma of rats with kidney ischemia-reperfusion injury [13,32]. The severity of the ischemia-reperfusion injury was increased in Nrf2 knockout mice [33]. Intraperitoneal injection of glutathione improved kidney function in Nrf2 knockout mice after ischemia-reperfusion injury [33]. Another study reported that activation of Nrf2 led to enhanced glutathione synthesis in the kidney of mice with ischemia-reperfusion injury [34]. A clinical trial revealed that pharmacological modulator bardoxolone methyl, a potent Nrf2 activator, alleviated kidney dysfunction in patients with CKD and type 2 diabetes [35].

The healthy kidneys prevent the urinary loss of folate through tubular reabsorption of this micronutrient [36]. Our recent study showed that kidney ischemia-reperfusion caused folate deficiency in rats due to decreased folate transporter expression and folate reabsorption in the kidney [37]. It is unclear if the administration of folic acid can improve kidney function impaired by AKI. The 5-MTHF is the primary form of folate found in blood and is biologically active. In the present study, we examined the effect of 5-MTHF on kidney injury in rats with ischemia-reperfusion-induced AKI. We investigated the mechanisms by which 5-MTHF attenuated oxidative stress in the kidney.

## 2. Materials and Methods

### 2.1. Induction of Kidney Ischemia-Reperfusion in Rats

Male Sprague-Dawley rats (6 weeks old, 250–300 g) were purchased from the University of Manitoba Central Animal Care Services (Winnipeg, MB, Canada). The rats were randomly divided into three groups: (1) kidney ischemia-reperfusion (IR), (2) kidney ischemia-reperfusion with folic acid (5-MTHF; Sigma Aldrich, Oakville, ON, Canada) injection (IR + 5-MTHF), and (3) sham-operated control (Sham) (*n* = 8 for each group). Kidney ischemia was induced by clamping the left renal pedicle with a non-traumatic vascular clamp for 45 min. At the end of ischemia, the clamp was removed to allow blood flow to the left kidney (reperfusion) and a right nephrectomy was performed as described in our previous studies [13,32,38,39]. In another group of rats, 5-MTHF (3 µg/kg body weight (BW)) was injected 30 min before ischemia and 3 h after reperfusion. The dose of 5-MTHF used in the present study was based on the results from a pilot study. The stock of 5-MTHF (2 mg/mL) was prepared and then diluted in saline for intraperitoneal (i.p.) injection in rats. The sham-operated group received a saline injection. A recent toxicity study revealed that daily oral administration of 5-MTHF (400 mg/kg) to rats for 13 weeks did not cause toxic effects on the kidneys and liver [40]. Rats with or without folic acid injection were sacrificed at 24 h after reperfusion. In the sham-operated group, rats received the same surgical procedure but without kidney ischemia and were sacrificed 24 h after the surgery. The kidney and blood were collected, and the plasma creatinine was measured by Cobas C111 Analyzer (Roche, Laval, QC, Canada). All procedures were performed in accordance with the Guide to the Care and Use of Experimental Animals published by the Canadian Council on Animal Care and approved by the University of Manitoba Protocol Management and Review Committee.

### 2.2. Biochemical and Histological Analysis

Malondialdehyde (MDA) was measured in the kidney and plasma using the thiobarbituric acid reactive substances (TBARS) method [32,41]. The reduced (GSH) and oxidized (GSSG) glutathione in the kidney were measured by a spectrophotometric detection method [32], and the ratio of GSH to GSSG was calculated. For histologic staining, the kidney was immersion-fixed in 10% neutral-buffered formalin, embedded in paraffin and sectioned (5 µm). The sections were stained with hematoxylin and eosin (H&E). The histological changes in the kidney were examined using Olympus BX43 light microscope (Olympus Corp, Tokyo, Japan) equipped with an Olympus Q-Color3 camera.

### 2.3. Cell Culture and siRNA Transfection

Human kidney proximal tubular cells (HK-2, ATCC, Manassas, VA, USA) were cultured at 37 °C in a normal atmosphere of 95% air and 5% CO_2_ in keratinocyte-serum free medium supplemented with human recombinant epidermal growth factor and bovine pituitary extract (Life Technologies, Carlsbad, CA, USA). Hypoxia-reoxygenation was induced in tubular cells to simulate ischemia-reperfusion injury [13,37,38]. In brief, hypoxia was induced in tubular cells by incubation for 2 h in a modified Krebs buffer (137 mM NaCl sodium chloride, 15.8 mM KCl potassium chloride, 0.49 mM MgCl_2_ magnesium chloride, 0.9 mM CaCl_2_ calcium chloride, 4 mM HEPES) supplemented with 10 mM 2-deoxyglucose, 20 mM sodium lactate, 12 mM KCl potassium chloride and 1 mM sodium dithionite (pH 6.4) in a hypoxia chamber (Billups-Rothenberg, Inc., Del Mar, CA, USA) containing 95% N_2_/5% CO_2_ at 37 °C. After 2 h hypoxia, the modified Krebs buffer was replaced with a keratinocyte-serum-free medium, and cells were cultured in 95% air and 5% CO_2_. To inhibit Nrf2 expression, tubular cells were transfected with human Nrf2 siRNA duplex oligoribonucleotides (30 pmoles, Stealth RNAi^TM^, Invitrogen, Carlsbad, CA, USA) by using Lipofectamine 2000 transfection reagent (Invitrogen). As a control, cells were transfected with Stealth^TM^ RNA negative control (Invitrogen) consisting of a scrambled. At 48 h after transfection, total mRNAs were prepared from cells. The mRNA of Nrf2 and antioxidant enzymes were determined. In another set of experiments, intracellular GSH was measured.

### 2.4. Quantitative Real-Time RT-PCR

Total RNA was prepared from the kidney tissue or tubular cells by using the TRIzol reagent (Invitrogen, Carlsbad, CA, USA). The mRNA of neutrophil gelatinase-associated lipocalin (NGAL), glutamate-cysteine ligase (Gclc, Gclm), glutathione synthetase (Gss), SOD-1, HO-1, Nrf2 and β-actin was determined by a real-time PCR analysis using the StepOnePlus^TM^ Real-Time PCR System (Applied Biosystems, Foster City, CA, USA) and normalized with β-actin. The primer sequences used in this study are presented in Table 1.

### 2.5. Western Immunoblotting Analysis

The protein levels of kidney glutamate-cysteine ligase (Gclc, Gclm), glutathione synthetase, SOD-1 and HO-1 were determined by Western immunoblotting analysis. Total proteins (10–60 µg) were separated by electrophoresis in 10% SDS polyacrylamide gels. Proteins in the gel were transferred to a nitrocellulose membrane. The membrane was probed with one of the following primary antibodies: rabbit anti-Gclc monoclonal (1:2000, Abcam, Inc., Toronto, ON, Canada), rabbit anti-Gclm monoclonal (1:2000, Abcam), rabbit anti-glutathione synthetase polyclonal (1:2000, Abcam), rabbit anti-SOD-1 polyclonal (1:2000, Abcam), or rabbit anti-HO-1 polyclonal (1:2000, Abcam) antibodies. This was followed by incubation of the membrane with HRP conjugated anti-rabbit IgG secondary antibodies (1:2000, Cell Signaling Technology, Danvers, MA, USA). Equal protein loading of each sample was confirmed by probing the nitrocellulose membrane with rabbit anti-β-actin monoclonal antibody (1:1000, Cell Signaling Technology). To determine the nuclear translocation of Nrf2, nuclear proteins (10–40 µg) were prepared from the kidney tissue. Nuclear proteins (40 µg) were separated by electrophoresis in 8% SDS polyacrylamide gels. The Nrf2 protein was detected by Western immunoblotting using anti-rabbit Nrf2 monoclonal antibodies (1:1000, Abcam). Equal protein loading of each sample was confirmed by probing the nitrocellulose membrane with rabbit anti-Lamin B1 polyclonal antibodies (1:1000, Abcam). The protein bands were visualized by using Luminata Crescendo chemiluminescent HRP detection reagent (Millipore Ltd., Etobicoke, ON, Canada).

### 2.6. Statistical Analysis

The statistical analysis was carried out using GraphPad Prism 8 for Windows (Version 8.0.2, GraphPad, San Diego, CA, USA). Results were analyzed using an unpaired two-tailed *t*-test or one-way ANOVA followed by a post-hoc analysis using the Newman–Keuls test according to test requirements. All the data were expressed as the mean ± SD. A statistical significance was considered when the *p*-value was less than 0.05.

## 3. Results

### 3.1. Folic Acid (5-MTHF) Improves Kidney Function

**Figure 1:**
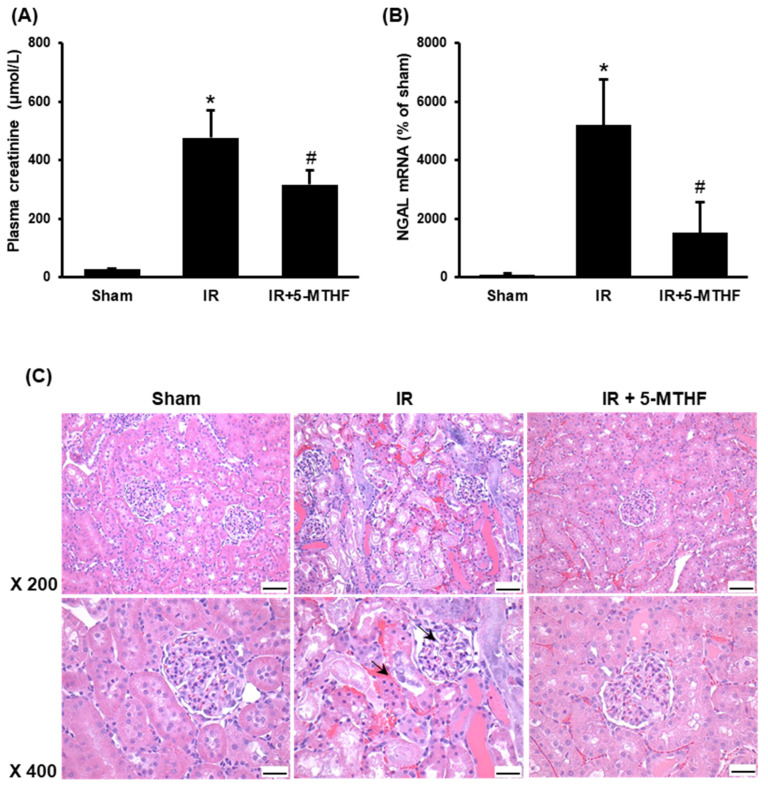
Kidney injury. Rats were subjected to sham operation (Sham), kidney ischemia-reperfusion (IR) or ischemia-reperfusion with 5-MTHF injection (IR + 5MTHF). (**A**) Plasma creatinine and (**B**) kidney NGAL mRNA were measured. Results are expressed as mean ± SD (*n* = 5–8). * *p* < 0.05 when compared with the value obtained from the Sham group. ^#^ *p* < 0.05 when compared with the value obtained from the IR group. (**C**) Representative hematoxylin and eosin (H&E) staining images of kidney sections are shown (magnification ×200, ×400). Arrows point to the area with red blood cell accumulation and irregular glomerular structure. The bar on the images represents 100 µm.

Ischemia-reperfusion caused a significant elevation of plasma creatinine levels, indicating a decline in kidney function (Figure 1A). Injection of 5-MTHF significantly reduced plasma creatinine levels in rats 24 h after ischemia-reperfusion injury (Figure 1A). An increased NGAL expression in the kidney is an early marker of proximal tubular injury. Ischemia-reperfusion caused a marked increase in NGAL mRNA expression while 5-MTHF injection significantly reduced NGAL mRNA in the kidneys of rats with ischemia-reperfusion injury (Figure 1B). Injection of 5-MTHF also improved kidney morphology in rats with ischemia-reperfusion injury (Figure 1C). These results suggested that 5-MTHF had a renal protective effect against ischemia-reperfusion injury.

### 3.2. Folic Acid (5-MTHF) Attenuates Ischemia-Reperfusion Induced Oxidative Stress

**Figure 2:**
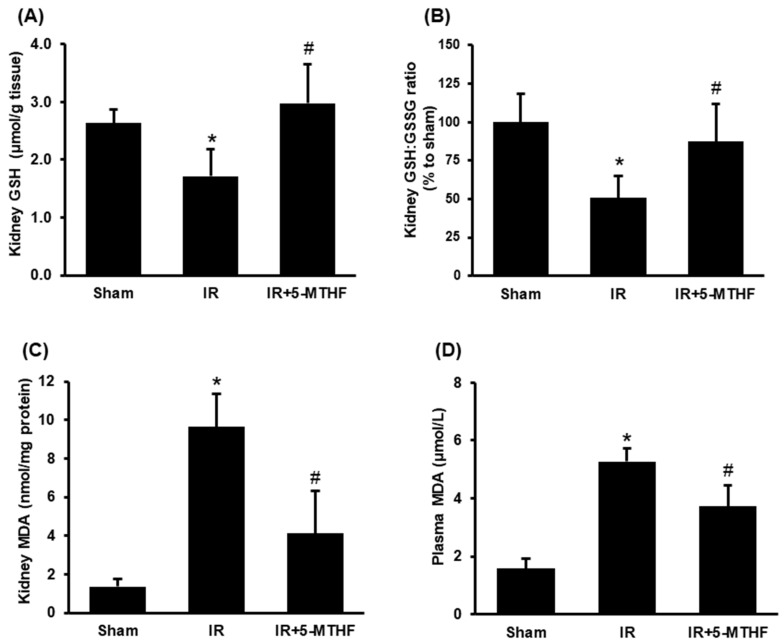
Oxidative stress biomarkers in the kidney. Rats were subjected to sham operation (Sham), kidney ischemia-reperfusion (IR) or ischemia-reperfusion with 5-MTHF injection (IR + 5-MTHF). (**A**) Glutathione (GSH) and (**B**) the ratio of GSH to GSSG were measured in the kidney. Malondialdehyde (MDA) in the kidney (**C**) and the plasma (**D**) were measured. Results are expressed as mean ± SD (*n* = 5–8). * *p* < 0.05 when compared with the value obtained from the Sham group. ^#^ *p* < 0.05 when compared with the value obtained from the IR group.

**Figure 3:**
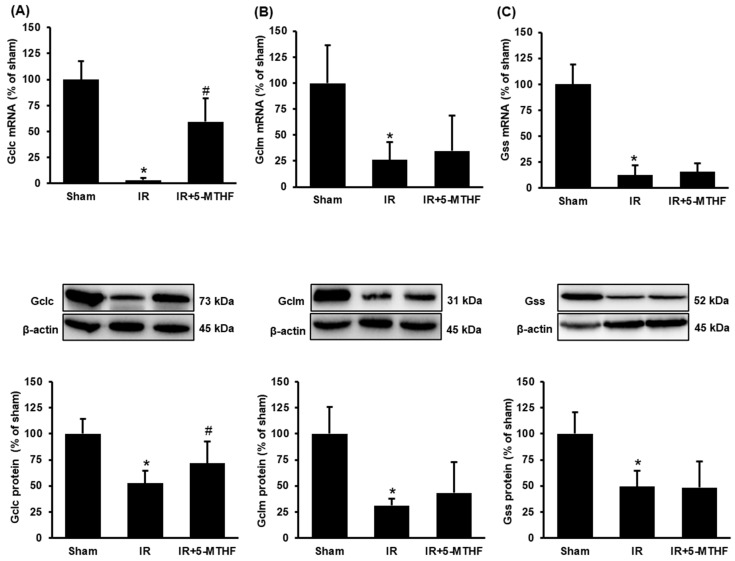
Glutathione-synthesizing enzyme expression in the kidney. Rats were subjected to sham operation (Sham), kidney ischemia-reperfusion (IR) or ischemia-reperfusion with 5-MTHF injection (IR + 5-MTHF). The mRNA and protein expression of (**A**) glutamate-cysteine ligase catalytic subunit (Gclc), (**B**) glutamate-cysteine ligase modifier subunit (Gclm) and (**C**) glutathione synthetase (Gss) were measured. Results are expressed as mean ± SD (*n* = 5–8). * *p* < 0.05 when compared with the value obtained from the Sham group. ^#^ *p* < 0.05 when compared with the value obtained from the IR group.

A reduction of GSH or the ratio of GSH to GSSG and an increase in malondialdehyde (MDA) are the indicators of oxidative stress. Ischemia-reperfusion caused a significant decrease in GSH level and the ratio of GSH to GSSG in the kidney (Figure 2A,B). Injection of 5-MTHF effectively restored the GSH level and the ratio of GSH to GSSG in the kidneys of rats with ischemia-reperfusion injury (Figure 2A,B). Ischemia-reperfusion caused a significant elevation of MDA in the kidney and plasma, indicating increased lipid peroxidation (Figure 2C,D). Injection of 5-MTHF significantly reduced lipid peroxidation in rats with ischemia-reperfusion injury (Figure 2C,D). To determine if folic acid modulated glutathione production, we measured the expression of enzymes responsible for glutathione synthesis. Ischemia-reperfusion injury significantly reduced the mRNA and protein levels of glutamate-cysteine ligase catalytic subunit (Gclc) and modifier subunit (Gclm), and glutathione synthetase in the kidney (Figure 3A–C). Injection of 5-MTHF significantly increased the expression of mRNA and protein levels of glutamate-cysteine ligase (Gclc) in the kidneys of rats with ischemia-reperfusion injury (Figure 3A); however, such treatment did not affect the expression of Gclm and glutathione synthetase (Figure 3B,C).

### 3.3. Folic Acid (5-MTHF) Stimulates Nrf2 and Antioxidant Enzyme Expression in the Kidney

**Figure 4:**
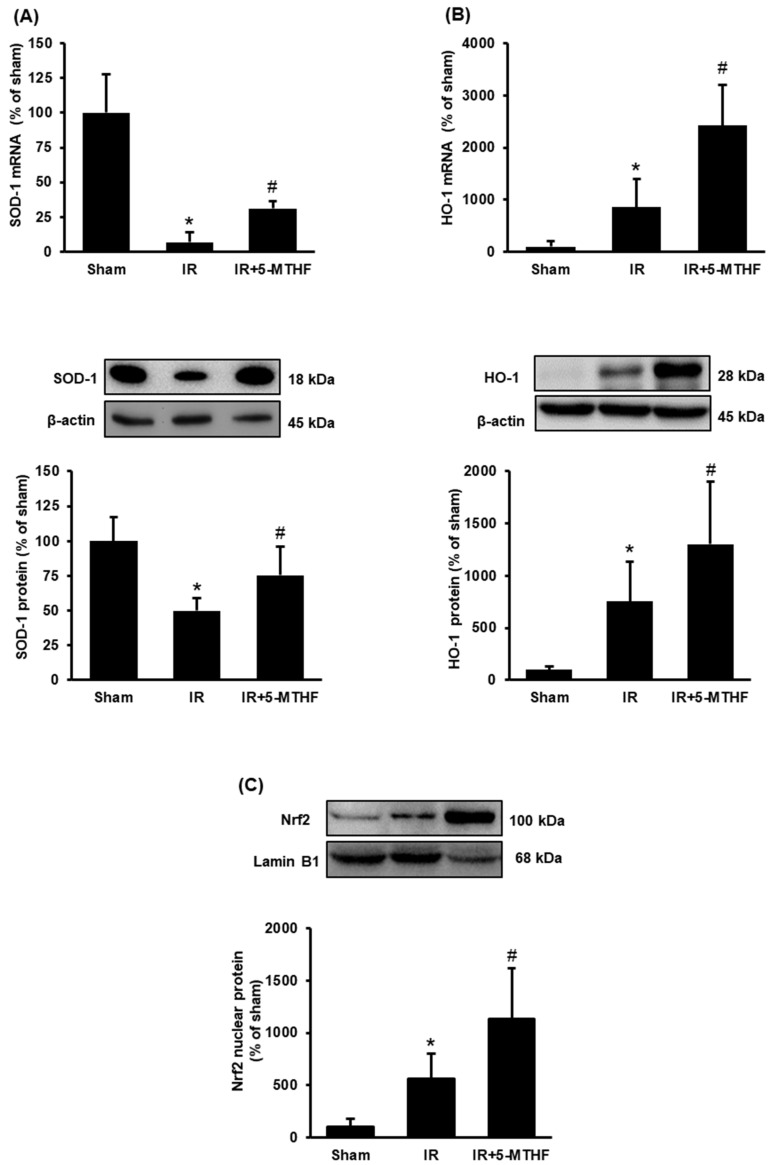
The expression of SOD-1 and HO-1 and Nrf2 in the kidney. Rats were subjected to sham operation (Sham), kidney ischemia-reperfusion (IR) or ischemia-reperfusion with 5-MTHF injection (IR + 5-MTHF). The mRNA and protein expression of (**A**) superoxide dismutase-1 (SOD-1), (**B**) heme oxygenase-1 (HO-1) and (**C**) nuclear Nrf2 protein were measured. Results are expressed as mean ± SD (*n* = 5–6). * *p* < 0.05 when compared with the value obtained from the Sham group. ^#^ *p* < 0.05 when compared with the value obtained from the IR group.

To investigate whether 5-MTHF affected Nrf2 signalling and antioxidant enzyme expression, we examined the expression of SOD-1, HO-1 and Nrf2 in the kidney. Aside from glutathione synthesizing enzymes, the expressions of SOD-1 and HO-1 are also regulated by Nrf2 in response to oxidative stress [27]. Ischemia-reperfusion significantly reduced the mRNA and protein levels of SOD-1 while 5-MTHF injection effectively restored SOD-1 expression to the level found in the sham-operated rats (Figure 4A). Although ischemia-reperfusion induced HO-1 expression, injection of 5-MTHF further augmented the elevation of HO-1 expression in the kidneys of rats with ischemia-reperfusion injury (Figure 4B). Ischemia-reperfusion increased the nuclear Nrf2 level in the kidneys, indicating the activation of Nrf2 (Figure 4C); however, injection of 5-MTHF caused a greater elevation of nuclear Nrf2 level in the kidneys of rats with ischemia-reperfusion injury (Figure 4C). These results suggested that 5-MTHF could further activate Nrf2 and increase antioxidant enzyme expression in the kidney upon ischemia-reperfusion injury.

### 3.4. Folic Acid (5-MTHF) Alleviates Cell Injury in Proximal Tubular Cells

**Figure 5:**
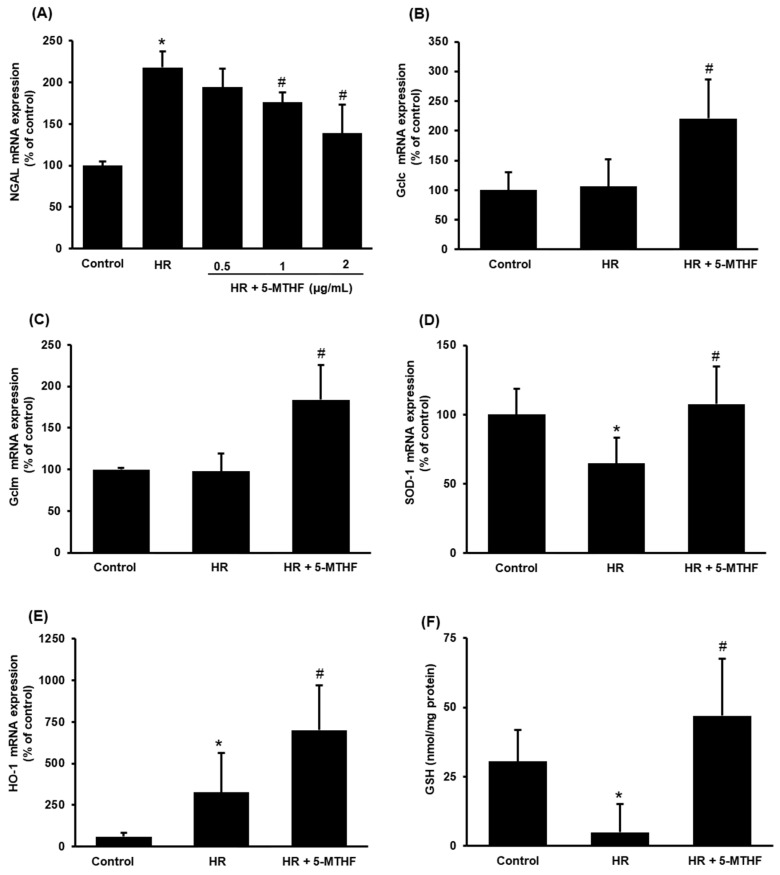
The expression of NGAL and glutathione synthesizing enzyme expression in proximal tubular cells. Tubular cells were subjected to 2 h hypoxia, followed by reoxygenation (HR) for 12 h or 24 h. In one set of cells, 5-MTHF (0.5,1 or 2 µg/mL) was added to the culture medium 30 min before hypoxia and during reoxygenation (HR + 5-MTHF). The mRNA of (**A**) neutrophil gelatinase-associated lipocalin (NGAL), (**B**) glutamate-cysteine ligase catalytic subunit (Gclc), (**C**) glutamate-cysteine ligase modifier subunit (Gclm), (**D**) superoxide dismutase-1 (SOD-1) and (**E**) heme oxygenase-1 (HO-1) were measured 12 h after hypoxia-reoxygenation. (**F**) Intracellular glutathione (GSH) was measured 24 h after hypoxia-reoxygenation. Results in (**B**–**F**) were obtained from cells incubated with 5-MTHF (2 µg/mL). Results are expressed as mean ± SD (*n* = 4–6). * *p* < 0.05 when compared with the value obtained from the sham group. ^#^ *p* < 0.05 when compared with the value obtained from the IR group.

To imitate the in vivo condition of ischemia-reperfusion, hypoxia-reoxygenation was applied to human proximal tubular cells. Hypoxia-reoxygenation caused a significant increase in NGAL expression, indicating tubular cell injury (Figure 5A). Preincubation of cells with 5-MTHF (1, 2 µg/mL) significantly reduced NGAL expression (Figure 5A). Incubation of cells with 5-MTHF also increased the expression of glutamate-cysteine ligase (Gclc, Gclm), SOD-1 and HO-1 in tubular cells after hypoxia-reoxygenation (Figure 5B–E). Furthermore, 5-MTHF significantly increased the intracellular glutathione level (Figure 5F). These results suggested that 5-MTHF could reduce hypoxia-reoxygenation-induced oxidative stress and alleviate injury in proximal tubular cells.

### 3.5. Inhibition of Nrf2 Expression Hampers Restoration of Glutathione Level and Antioxidant Enzyme Expression by Folic Acid (5-MTHF)

**Figure 6:**
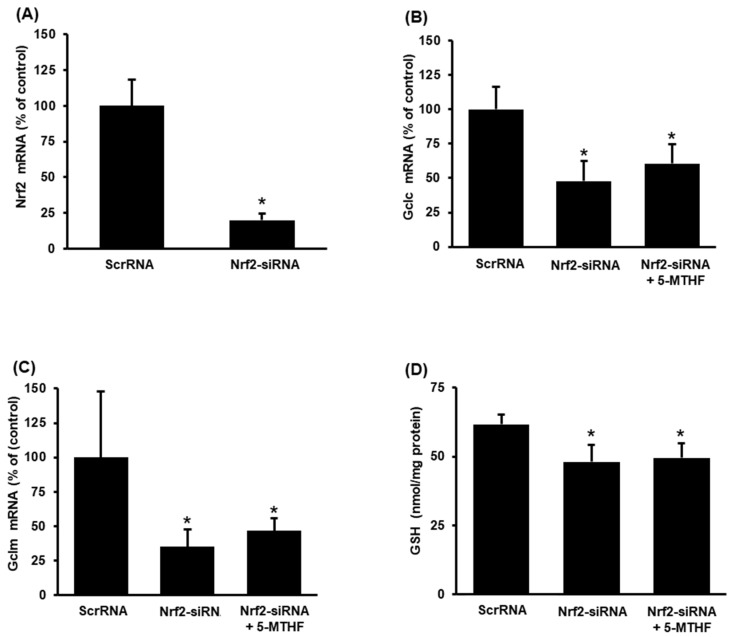
Nrf-2 siRNA transfection in proximal tubular cells. Tubular cells were transfected with Nrf2 siRNA or scrambled RNA (ScrRNA) as a control followed by hypoxia-reoxygenation. In one set of cells, 5-MTHF (2 µg/mL) was added to the culture medium 30 min before hypoxia and during reoxygenation. The mRNA of (**A**) Nrf2, (**B**) glutamate-cysteine ligase catalytic subunit (Gclc) and (**C**) glutamate-cysteine ligase modifier subunit (Gclm) was measured 12 h after hypoxia-reoxygenation. (**D**) The intracellular glutathione (GSH) was measured 24 h after hypoxia-reoxygenation. Results are expressed as mean ± SD (*n* = 4). * *p* < 0.05 when compared with the value obtained from cells transfected with ScrRNA.

To confirm the involvement of Nrf2 signalling in folic acid action, Nrf2 expression was inhibited by siRNA silencing. The Nrf2 siRNA transfection effectively inhibited Nrf2 expression in tubular cells compared to that in control cells that were transfected with scrambled siRNA (Figure 6A). Inhibition of Nrf2 expression abolished the stimulatory effect of 5-MTHF on glutamate-cysteine ligase (Gclc, Gclm) expression (Figure 6B,C). The Nrf2 inhibition also prevented the restoration of intracellular glutathione levels by 5-MTHF in tubular cells with hypoxia-reoxygenation (Figure 6D).

## 4. Discussion

Oxidative stress is one of the major factors mediating adverse outcomes in patients with AKI [42]. Antioxidants have been implicated in the mitigation of kidney injury in early AKI [43]. The deficiency of micronutrients, such as folate, is found in patients with kidney diseases [19,20]. The present study, for the first time, demonstrated that injection of a low-dose biologically active form of folate (5-MTHF) could attenuate oxidative stress and improve kidney function in rats with ischemia-reperfusion-induced AKI. Further investigation in human proximal tubular cells identified that the antioxidative effect of 5-MTHF was mediated, in part, through Nrf2 activation, increased antioxidant enzyme expression and glutathione level in the kidneys and proximal tubular cells. It is plausible that a folic acid supplement may improve outcomes in AKI.

Oxidative stress is an unavoidable consequence of ischemia-reperfusion injury, a major risk factor for AKI. It arises from an imbalance between reactive free radical generation and the antioxidant defence system in the body [10]. Oxidative stress is associated with worse clinical outcomes in patients with kidney diseases [11]. In line with the findings from clinical and animal studies, we observed that rats with ischemia-reperfusion had severely impaired kidney function and increased oxidative stress. Targeting oxidative stress is emerging as a promising strategy for the prevention and treatment of AKI and other diseases [14,18,44]. Folate (folic acid) is an essential micronutrient for DNA synthesis and one-carbon reactions in mammalian cells. Previous studies have shown the antioxidative effect of folic acid in patients and animal models [3,45]. Folate deficiency happens in patients with kidney transplants and in rats with ischemia-reperfusion injury [19,37]. In the present study, injection of low-dose 5-MTHF, a biologically active form of folate, into rats significantly improved kidney function and decreased oxidative stress biomarkers in rats with kidney ischemia-reperfusion injury. Glutathione is a major endogenous non-enzymatic antioxidant in the body. Its depletion is associated with impaired kidney function in patients with AKI [46]. In the present study, ischemia-reperfusion caused a significant decrease in glutathione synthesizing enzyme expression and glutathione level in the kidney. Injection of 5-MTHF partially restored the expression of glutamate-cysteine ligase (Gclc), an enzyme that regulates the rate-limiting step in glutathione synthesis [29]. This was accompanied by a significant elevation of GSH level and a ratio of GSH to GSSG in the kidneys of rats with ischemia-reperfusion injury. Furthermore, 5-MTHF could prevent cell injury and restore glutathione levels in proximal tubular cells with hypoxia-reoxygenation insult. The intracellular GSH content represents the dynamic balance between GSH synthesis, rate of its consumption and efflux, which regulate the intracellular GSH concentrations [47]. Our results suggested that hypoxia-reoxygenation-induced oxidative stress might have depleted GSH in proximal tubular cells. The 5-MTHF significantly increased the expression of the rate-limiting enzyme Gclc and Gclm in cells challenged with hypoxia-reoxygenation, which, in turn, restored intracellular GSH levels. These results suggested that restoration of glutathione synthesis might contribute, in part, to the antioxidative and renal protective effect of folic acid against ischemia-reperfusion injury.

The transcription factor Nrf2 is an important regulator of the antioxidant defence system. Activation of Nrf2 induces the expression of antioxidant enzymes to maintain a redox balance in the body. Under physiological conditions, Nrf2 is bound with a repressor protein Keap1 in the cytoplasm. Upon oxidative stress, Nrf2 is disassociated from Keap1 and is translocated into the nucleus where it activates the expression of genes encoding antioxidant enzymes [26,27,48]. Genetic deletion of Nrf2/keap-1 resulted in kidney dysfunction and increased oxidative stress in mice subjected to ischemia-reperfusion injury [33,34]. In the present study, ischemia-reperfusion significantly increased nuclear Nrf2 levels but failed to stimulate the expression of SOD-1 and glutathione synthesizing enzymes in the kidney. These results suggested that although ischemia-reperfusion elevated nuclear Nrf2 levels, such an adaptive regulation of antioxidant defence might not be sufficient to overcome increased oxidative stress caused by ischemia-reperfusion injury. In contrast, injection of 5-MTHF at a low dosage could further activate Nrf2 in the kidneys of rats after ischemia-reperfusion injury, leading to restoration of glutathione levels; injection of 5-MTHF also effectively restored SOD-1 expression and further increased HO-1 expression in the kidneys of rats with ischemia-reperfusion injury. In addition, gene silencing in human proximal tubular cells further established the role of Nrf2 in mediating the antioxidative effect of 5-MTHF against ischemia-reperfusion injury. In tubular cells with Nrf2 gene silencing, 5-MTHF could no longer stimulate the expression of antioxidant enzymes or elevate intracellular glutathione levels upon hypoxia-reoxygenation. Taken together, these results suggested that folic acid could activate Nrf2 and hence stimulate the expression of antioxidant enzymes in the kidney. Mitigation of oxidative stress, in turn, might improve kidney function after ischemia-reperfusion-induced AKI.

Elevation of oxidative stress biomarkers is strongly associated with a reduction of antioxidant status in patients with kidney disease [11,49]. The KDIGO guidelines for AKI management are mainly focused on improving metabolic complications in patients with AKI [16]. Malnutrition remains the major concern in AKI patients with micronutrient deficiency [16,17,20]. As a micronutrient, folate/folic acid has diverse biological functions including its antioxidative effect [2,3]. We recently reported that ischemia-reperfusion impaired folate transporter (FOLR1) expression in the kidney leading to decreased folate reabsorption and low circulating folate levels in rats [37]. The deficiency of micronutrients including folate is common in patients with AKI [19,20]. Recommended Dietary Allowance (RDA) for dietary folate intake is 400 μg per day for adults [50]. Although daily oral intake of folic acid (5–15 mg) did not show adverse effects in healthy people [51], folic acid at very high doses can be renal toxic. It was reported that single or multiple injections of high-dose folic acid (125–250 mg/kg BW) caused tubular necrosis, oxidative stress and increased creatinine levels in rodents [52,53,54]. On the other hand, low-dose folic acid had beneficial effects on the cardiovascular system, and improved hyperhomocysteinemia and diabetes [23,45,55,56]. The 5-MTHF is biologically active and is the primary form of folate in systemic circulation. In the present study, injection of low-dose 5-MTHF significantly reduced oxidative stress biomarkers and improved kidney function in rats 24 h after ischemia-reperfusion injury. The initial injury to proximal tubular cells can occur as early as ischemia and the early phase of reperfusion. In the present study, the first dose of 5-MTHF was injected 30 min before ischemia to provide initial renal protection. The second dose was injected 3 h after ischemia, which might exert substantial effects on the renal protection by maintaining sufficient 5-MTHF levels in the circulation. To the best of our knowledge, we are the first to report that injection of the biologically active form of folate (5-MTHF) could attenuate oxidative stress and improve kidney function in rats with ischemia-reperfusion-induced AKI. Future studies are warranted to identify the optimal dose range of 5-MTHF and its long-term impact on kidney function preservation. Furthermore, the clinical application of such an approach remains to be validated in patients with kidney disease.

## 5. Conclusions

In conclusion, the present study has demonstrated that injection of low-dose biologically active form of folic acid (5-MTHF) can attenuate oxidative stress and improve kidney function in rats with ischemia-reperfusion-induced AKI. The antioxidant effect of folic acid is mediated, in part, through activation of Nrf2, the elevation of antioxidant enzyme expression and restoration of glutathione level in the kidney. Results from the present study may provide a new perspective on the use of micronutrient supplements against oxidative stress and their implication in improving clinical outcomes in patients with kidney diseases.

## Figures and Tables

**Table 1:** Gene primer sequences of rat and human used for real-time PCR.

Target Gene	Forward Primer (5′-3′)	Reverse Primer (5′-3′)	Accession Number
Rat			
NGAL	GATCAGAACATTCGTTCCAA	TTGCACATCGTAGCTCTGTA	NM_130741.1
Gclc	GCCCAATTGTTATGGCTTTG	AGTCCTCTCTCCTCCCGTGT	NM_012815.2
Gclm	CGAGGAGCTTCGAGACTGTAT	ACTGCATGGGACATGGTACA	NM_017305.2
Gss	ACAACGAGCGAGTTGGGAT	TGAGGGGAAGAGCGTGAATG	NM_012962.1
SOD-1	CATTCCATCATTGGCCGTACT	CCACCTTTGCCCAAGTCATC	NM_017050.1
HO-1	CGACAGCATGTCCCAGGATT	TCGCTCTATCTCCTCTTCCAGG	NM_012580.2
β-actin	ACAACCTTCTTGCAGCTCCTC	GACCCATACCCACCATCACA	NM_031144.3
Human			
NGAL	GAAGACAAAGACCCGCAAAAG	CTGGCAACCTGGAACAAAAG	NM_005564.5
Gclc	TACAGTTGAGGCCAACATGC	CTTGTTAAGGTACTGGGAAATGAG	NM_001197115.2
Gclm	GTTCAGTCCTTGGAGTTGCACA	CCCAGTAAGGCTGTAAATGCTC	NM_001308253.2
SOD-1	CTCACTCTCAGGAGACCATTGC	CCACAAGCCAAACGACTTCCAG	NM_000454.5
HO-1	CCAGGCAGAGAATGCTGAGTTC	AAGACTGGGCTCTCCTTGTTGC	NM_002133.3
β-actin	AGATCAAGATCATTGCTCCTCCT	GATCCACATCTGCTGGAAGG	NM_001101.5

## Data Availability

Data is contained within the article.
